# Recent Advances and Future Perspectives in Polymer-Based Nanovaccines

**DOI:** 10.3390/vaccines9060558

**Published:** 2021-05-26

**Authors:** Natassa Pippa, Maria Gazouli, Stergios Pispas

**Affiliations:** 1 Theoretical and Physical Chemistry Institute, National Hellenic Research Foundation, 48 Vassileos Constantinou Avenue, 11635 Athens, Greece; natpippa@pharm.uoa.gr; 2 Department of Pharmaceutical Technology, Faculty of Pharmacy, Panepistimioupolis Zografou, National and Kapodistrian University of Athens, 15771 Athens, Greece; 3 Department of Basic Medical Science, Laboratory of Biology, School of Medicine National, Kapodistrian University of Athens, 11527 Athens, Greece; mgazouli@med.uoa.gr; 4 Department of Radiology, University General Hospital “Attikon”, School of Medicine, National and Kapodistrian University of Athens, 11527 Athens, Greece

**Keywords:** polymers, biodegradable polymers, poly(lactic-co-glycolic acid) (PLGA), nanoparticles, nanovaccines, adjuvants, antigen-presenting cells (APCs), depot effect, antigens

## Abstract

Vaccination is the most valuable and cost-effective health measure to prevent and control the spread of infectious diseases. A significant number of infectious diseases and chronic disorders are still not preventable by existing vaccination schemes; therefore, new-generation vaccines are needed. Novel technologies such as nanoparticulate systems and adjuvants can enable safe and effective vaccines for difficult target populations such as newborns, elderly, and the immune-compromised. More recently, polymer-based particles have found application as vaccine platforms and vaccine adjuvants due to their ability to prevent antigen degradation and clearance, coupled with enhanced uptake by professional antigen-presenting cells (APCs). Polymeric nanoparticles have been applied in vaccine delivery, showing significant adjuvant effects as they can easily be taken up by APCs. In other words, polymer-based systems offer a lot of advantages, including versatility and flexibility in the design process, the ability to incorporate a range of immunomodulators/antigens, mimicking infection in different ways, and acting as a depot, thereby persisting long enough to generate adaptive immune responses. The aim of this review is to summarize the properties, the characteristics, the added value, and the limitations of the polymer-based nanovaccines, as well as the process of their development by the pharmaceutical industry.

## 1. Introduction

Vaccination is the most valuable and cost-effective health measure to prevent and control the spread of viral/bacterial infectious diseases responsible for high mortality and morbidity. According to the World Health Organization [1]: “Vaccines reduce risks of getting a disease by working with your body’s natural defences to build protection. When you get a vaccine, your immune system responds. It recognizes the invading germ, such as the virus or bacteria; produces antibodies and remembers the disease and how to fight it. If you are then exposed to the germ in the future, your immune system can quickly destroy it before you become unwell. The vaccine is therefore a safe and clever way to produce an immune response in the body, without causing illness. Our immune systems are designed to remember. Once exposed to one or more doses of a vaccine, we typically remain protected against a disease for years, decades or even a lifetime. This is what makes vaccines so effective. Rather than treating a disease after it occurs, vaccines prevent us in the first instance from getting sick.”

A significant number of infectious diseases and chronic disorders such as HIV, tuberculosis, malaria, healthcare-associated infections (HAIs), cytomegalovirus (CMV), and respiratory syncytial virus (RSV) are still not preventable by vaccination and require new-generation vaccines. According to Strategic Research Agenda for Innovative Medicines Initiative [2]: “Novel technologies such as adjuvants (including immunomodulators and molecular targeting), new vectors, cell-based vaccines and new devices can also enable effective vaccines for difficult target populations such as new-borns, elderly and the immune compromised and could help developing effective therapeutic vaccines targeting not only infectious diseases but also cancer and other chronic disorders.”

**Figure 1:**
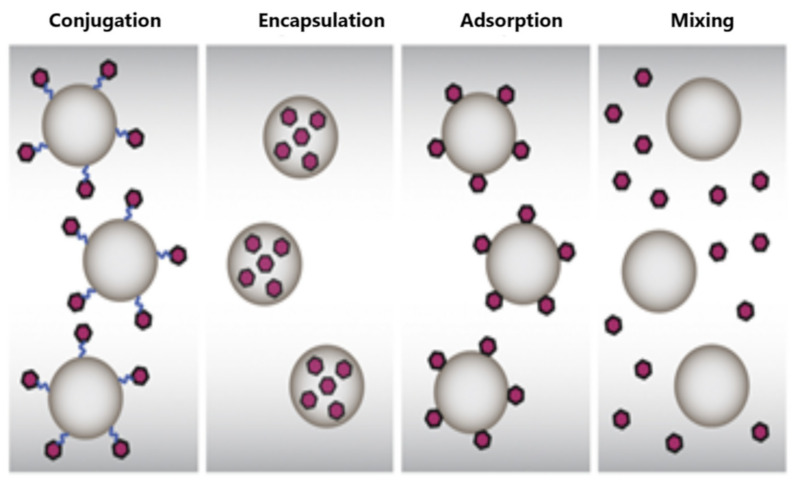
Interaction of nanoparticles with an antigen of interest. Formulation of nanoparticle and antigen of interest can be implemented through attachment (e.g., conjugation, encapsulation, or adsorption) or simple mixing. (Adapted from [5]).

Nanoparticles composed of biomimetic immunomodulatory materials have been characterized as unique delivery carriers and adjuvants for vaccine application [2,3,4,5,6,7,8,9,10,11,12,13,14]. Several nanocarriers and nanovectors have appeared in the literature for antigen/protein delivery and/or with adjuvant properties [2,3,4,5,6,7,8,9,10,11,12,13,14]. Liposomes, virus-like particles, inorganic nanoparticles (nanotubes, mesoporous spheres, gold nanoparticles), polymer-based systems, emulsions, and dendrimers are the main systems of pharmaceutical nanotechnology used for the design and the development of nanovaccines [2,3,4,5,6,7,8,9,10,11,12,13,14]. Nanomaterials exhibit several advantages that make them ideal as innovative platforms for vaccine applications [2,3,4,5,6,7,8,9,10]. The loading/encapsulation efficiency of antigens (proteins/peptides) is very high. They also improve their stability in vitro and in vivo. Targeting (site-specific and/or temporal) can also be achieved [2,3,4,5,6,7,8,9,10]. Different mechanisms have been used for the loading of antigen as well. Figure 1 illustrates the interaction of nanoparticles with an antigen of interest. The main mechanisms are conjugation, encapsulation, adsorption, or simple mixing [5]. Last but not least, the added value of nanosystems for vaccines is the programming of the immune responses. This is a very useful design strategy not only for the infectious disease vaccines but also for cancer vaccines [2,3,4,5,6,7,8,9,10,11,12,13,14].

**Scheme 1:**
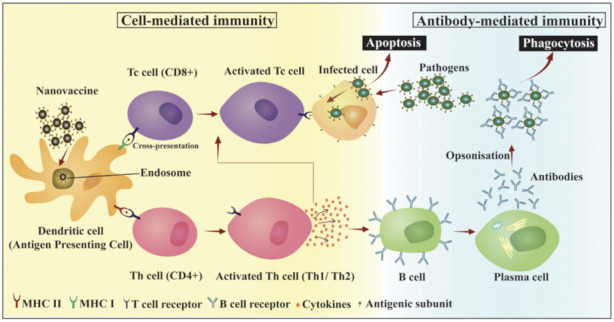
Activation of adaptive immunity by nanovaccines: uptake and presentation of antigenic subunit by APCs elicit cell-mediated and antibody-mediated immune response leading to apoptosis of infected cells and phagocytosis of antibody–pathogen complex. (Adapted from [6]).

More specifically, polymer-based particles have found several applications as vaccine platforms and adjuvants due to their ability to prevent antigen degradation and clearance, with enhanced uptake by professional antigen-presenting cells (APCs) [2,3,4,5,6,7,8,9,10,11,12,13,14]. Polymeric nanoparticles have been applied in vaccine delivery, showing significant adjuvant effects as they can easily be taken up by antigen-presenting cells. In other words, polymer-based systems offer a lot of advantages, including versatility and flexibility in the design process, the ability to incorporate a range of immunomodulators/antigens, mimicking infection in different ways, and acting as a depot, thereby persisting long enough to generate adaptive immune responses [3,4,5,6,7,8,9,10]. Scheme 1 presents the activation of adaptive immunity by nanovaccines: uptake and presentation of antigenic subunit by APCs elicit cell-mediated and antibody-mediated immune response leading to apoptosis of infected cells and phagocytosis of antibody–pathogen complex. Figure 1 shows the interaction of polymer nanoparticles with antigen of interest. Formulation of nanoparticle and antigen of interest can be implemented through attachment (e.g., conjugation, encapsulation, or adsorption) or simple mixing The aim of this review is to summarize several examples, the properties, the characteristics, the added value, and the limitations of the polymer-based nanovaccines, as well as the process of their development by the pharmaceutical industry.

## 2. Methodology of Literature Review

Systematic search and review of papers regarding polymer-based nanovaccines took place via MedLine, Scopus, and Web of Science platforms and abstract presentations of international conferences.

## 3. The Application of Polymers in Nanovaccines

### 3.1. Polymer-Based Nanovaccines

Polymers have been extensively studied as components and excipients for vaccine platforms in the immunotherapy of various infectious diseases, immunotherapy, and cancer. Polylactide-co-glycolide (PLGA) polymer-based nanosystems are one example—the most famous—with numerous literature references. PLGA is a biocompatible and biodegradable polymer material because it is metabolized in the human body by enzymes in monomers of the lactic acid and the glycolic acid. PLGA polymer can self-assemble into different morphologies at nano- or micro-scale, which are strongly dependent on the preparation method, the aqueous medium, and the other components of the formulation [11,12,13,14,15,16,17,18,19,20,21,22,23,24,25,26,27,28,29,30,31,32,33,34,35,36,37,38,39,40,41,42,43,44,45,46,47,48,49,50,51,52,53,54,55,56,57,58,59,60,61,62,63,64,65,66]. Namely, the PLGA formulations that are used as vaccine (and/or drug) delivery platforms are (functionalized) nanoparticles, nanospheres, nanoemulsions, micelles, and (nano/hydro)gels. [11,12,13,14,15,16,17,18,19,20,21,22,23,24,25,26,27,28,29,30,31,32,33,34,35,36,37,38,39,40,41,42,43,44,45,46,47,48,49,50,51,52,53,54,55,56,57,58,59,60,61,62,63,64,65,66]. The physicochemical characteristics, the solubility, and the thermodynamic/physicochemical stability of PLGA nanosystems can be fine-tuned extensively. Further, PLGA can be conjugated with polyethylene glycol (PEG) or polyetherimide to form block copolymers, which can self-assemble into polymeric micelles, and the resulting micellar nanoparticles can incorporate hydrophobic molecules and hydrophobic peptide antigens or proteins [11,12,13,14]. Table 1 summarizes the different types of nanoparticles, their characteristics, and their disadvantages. We present and analyze different examples from the recent literature.

Porous poly(lactic-co-glycolic acid) (PLGA) and poly(L-lactic acid) (PLA) nanoparticles have been investigated for pulmonary delivery of hepatitis B vaccine [15]. Three different formulations of PLA and PLGA nanoparticles containing a standard amount of hepatitis B surface antigen (HBsAg) were designed, developed, and prepared by a double-emulsion-solvent-evaporation method. The immune responses were studied by quantitating the secretion of IgA in fluids of mucosa and measuring cytokine levels in mice spleen homogenates. The nanoparticle hydrophilicity/hydrophobicity on mucosal and cell-mediated immune responses was also investigated. Namely, the hydrophobic nanoparticles with a size larger than 500 nm elicited a more robust increase in IgA, interleukin-2, and interferon-γ levels compared to hydrophilic nanoparticles with a size smaller than 500 nm. According to the described results the prepared inhalable polymeric nanoparticles of HBsAg exhibit an enhancement of immune responses [15]. In other words, the prepared aerosolized and inhaled PLA and PLGA nanoparticles enhance the responses (humoral, mucosal, and cytokine) to hepatitis B vaccine [15].

Diwan et al. investigated the co-delivery of CpG synthetic oligodeoxynucleotides and antigens in biodegradable nanospheres as an alternative approach for immunization, using tetanus toxoid as the model antigen and oligodeoxynucleotide (ODN) #1826 as the model CpG sequence. The results suggested that the co-delivery of CpG ODN adjuvants and antigens in nanospheres is a more efficient delivery approach for immunization than the use of the antigens alone in dispersion state [16]. Immune response by nasal delivery of hepatitis B surface antigen and codelivery of a CpG ODN in alginate-coated chitosan nanoparticles was also achieved by Borges et al. [19]. Alginate-coated chitosan nanoparticles were loaded with the recombinant hepatitis B surface antigen (HBsAg) and applied to mice by the intranasal route. All intranasally vaccinated groups showed higher interferon-γ secretion when compared to naive mice [16].

Poly(lactide-co-glycolide) (PLGA) nanoparticles were used for the delivery of a stable immunogenic domain 4 of protective antigen (PAD4) of Bacillus in order to overcome the issues of dosage, nanotoxicity of adjuvant, and the limited stability associated with anthrax vaccines according to a recent publication [17]. The nanoformulations were prepared by water/oil/water solvent evaporation method. The PAD4 systems induced an IgG response with mixed IgG1 and IgG2a subtypes, whereas the control PAD4-immunized mice elicited low IgG response with predominant IgG1 subtype. The PAD4 systems also induced both Th1 and Th2 responses, whereas PAD4 elicited predominantly Th2 response [17]. The effectiveness and the efficacy of this single-dose vaccine nanoformulation were compared with those of the recombinant PAD4 in providing protective immune response against a lethal challenge with Bacillus anthracis spores; the median survival of PAD4-NP-immunized mice was 6 days as compared to 1 day for PAD4-immunized mice [17].

According to Lima et al., mice treated with viable Mycobacterium tuberculosis with no glycolipid trehalose dimycolate (TDM) on the outer cell wall (delipidated Mycobacterium tuberculosis) by intraperitoneal and intratracheal inoculation presented intense recruitment of polymorphonuclear cells into the peritoneal cavity and acute inflammatory reaction in the lungs, respectively [18]. TDM-loaded biodegradable PLGA microspherical particles as well as TDM-coated charcoal particles induced an inflammatory reaction. Microspheres were prepared using the emulsion solvent evaporation technique. In addition, high levels of interleukin-6 (IL-6), tumor necrosis factor-alpha (TNF-alpha), IL-12, IL-10, interferon-γ, and IL-4 production were detected in lung cells, and nitric oxide (NO) production was high in culture supernatants of bronchoalveolar lavage cells [18,19].

Alginate–poly(ethylenimine) (PEI) is a bio-reducible polymer material self-assembled into nanogel formulation for antigen loading and delivery vehicle that significantly improves vaccine-elicited humoral and cellular immune responses [20]. The alginate–poly(ethylenimine) nanogels were formulated by the well-known technique of the electrostatic interaction of negatively charged sodium alginate with branched bioreducible cationic PEI followed by disulfide cross-linking to formulate bioreducible nanogels [20]. This nanoplatform ameliorates vaccine-induced antibody secretion and CD8^+^ T-cell-mediated tumor cell lysis. For this reason, this polymer vehicle could serve as a potent adjuvant system to improve vaccine-elicited humoral and cellular immune responses [20].

Hasegawa et al. developed a complex system composed of cholesterol-bearing hydrophobized pullulan and the protein NY-ESO-1 [21]. This protein belongs to a class of cancer/testis antigens and has been investigated as an immunogenic molecule in patients with different cancer types. From the in vitro experiments, the stimulation of CD8 and CD4 T cells from peripheral blood mononuclear cells in healthy volunteers with autologous of cholesterol-bearing hydrophobized pullulan/ESO-loaded dendritic cells as APCs were also investigated. The results were very promising for the development of a polyvalent cancer vaccine [21].

Saad et al. showed the ability of Advax adjuvant, a novel polysaccharide adjuvant based on delta inulin, to enhance the immunogenicity of hepatitis B surface antigens (HBs) in mice and guinea pigs in comparison to the traditional alum adjuvant [22]. Enhanced immune response and protective effects of nanochitosan-based DNA vaccine encoding T cell epitopes of Esat-6 and FL against Mycobacterium tuberculosis infection have also appeared in the literature [23]. The immunized mice remarkably elicited enhanced T-cell responses and protection against Mycobacterium tuberculosis challenge [23]. A hyperbranched polyglycerol multifunctionalized by “click chemistry” was synthesized, and a tumor-associated MUC1 glycopeptide combined with the immunostimulant T-cell epitope P2 from tetanus toxoid was loaded [24]. This globular polymeric system exhibited a flexible dendrimer-like morphology, which allowed optimal antigen presentation to the immune system and strong immune responses in mice and IgG antibodies recognizing human breast-cancer cells [24].

Poly(ethylene glycol)-b-poly(L-lysine)-b-poly(L-leucine) (PEG-PLL-PLLeu) polypeptides were self-assembled into micelles with significant cationic surface charge. The aforementioned hybrid polypeptides were designed as vaccine delivery platforms [25]. The authors proved that the prepared polypeptide cationic micellar formulations robustly enhanced vaccine-induced antibody secretion by 70–90-fold, which could be due to their capability of inducing different biological pathways of the immune system (i.e., dendritic cell maturation, improving antigen uptake and presentation to APCs, promoting germinal center formation) [25].

Zhang et al. formulated an “easy-to-adopt” strategy to enhance immune responses using functionalized alginate nanoparticles. The functionalized alginate nanoparticles were prepared by cross-linking of two different types of alginate using CaCl_2_ [26]. The mannose modified alginate was utilized for the specific targeting to the DCs. The authors also used ovalbumin (OVA) as model antigen and conjugated it to alginate molecules via the mechanism of pH-sensitive Schiff base bond. The above-described delivery platform was studied as a potential vaccine for cancer immunotherapy because it was found to increase the cross-presentation of OVA to B3Z T-cell hybridoma in vitro [27]. The subcutaneous administration of this nanovaccine also induced a strong cytotoxic T-lymphocyte response and the parallel inhibition of E.G7 tumor growth in C57BL/6 mice [26]. These pH-responsive alginate nanoparticles exhibit an added value to cancer immunotherapy due to spatiotemporal control of the incorporated antigen [27]. According to Démoulis et al., alginate-coated chitosan nanogel has the ability to control the effect of toll-like receptor (TLR) ligands on blood dendritic cells [28]. The findings of the experimental procedure showed that the influence of alginate-coated chitosan nanogels on human blood DC endocytosis of the TLR ligands was apparently a major contributory element. The last observation demonstrates the significance of predefining the interplay between delivery platforms and the immunostimulatory compounds for ensuring appropriate immune activation and efficacious combinations [28]. The same group prepared alginate-coated chitosan nanogel formulations for the encapsulation of CpG-oligodeoxynucleotides (class-A or class-B CpG-ODNs). The results of the immune response of these platforms of nanogels were compared with the same free CpG-ODNs or with pure nanogels [29]. Experiments were performed on both porcine and human blood DC subpopulations. Incorporation of class-A CpG-ODN into alginate nanogels significantly reduced the CpG-ODN uptake and intracellular trafficking in the cytosol. On the contrary, incorporation of class-B CpG-ODN increased its uptake and did not consistently influence intracellular trafficking into the nucleus. The selection of the CpG-ODN system as an adjuvant form is thus very important in terms of how it will behave with nanoparticulate vaccine delivery systems, exhibiting distinctive modulatory influences on the CpG-ODN [29].

#### 3.1.1. Micelles

**Figure 2:**
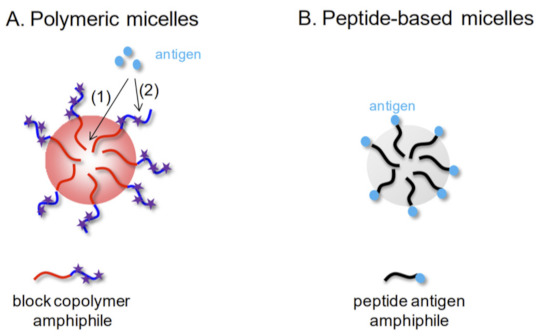
Schematical representation of the micellar nanoparticles developed as vaccine adjuvants: (**A**) polymeric micelles obtained by self-assembly of amphiphilic block copolymers in water, in/on which the antigenic peptide is encapsulated (1) or surface-coupled (2) (reactive groups represented by star symbols); (**B**) micelles obtained from self-assembly of peptide antigen amphiphiles in water. (Adapted from [30]).

Self-assembled micellar nanoparticles from amphiphilic biomacromolecules have been characterized as an innovative strategy to improve the efficacy of vaccines and subunit vaccines [30]. Figure 2 represents in detail the micellar nanoparticles designed and developed as vaccine adjuvants. The two main types of micellar nanoparticles are based on polymers or on peptides. Polymeric micelles are obtained by self-assembly of amphiphilic block copolymers in aqueous or buffer media, in/on which the antigenic peptide is incorporated or attached by chemical reactions onto surface. The peptide-based micelles are also obtained from self-assembly of peptide antigen amphiphiles in water media [30].

Their numerous advantages are their ease of formulation and scale-up, low size (enabling entering in lymphatic capillaries for reaching lymph nodes), size/surface tunability, surface modification, and chemical versatility enabling introduction of stimuli (e.g., pH, temperature, light)-responsive features and biofunctionalization with specific compounds and molecules [31].

Luo et al. designed, prepared, and evaluated a polyethylene glycol-b-poly ϵ-caprolactone-g-polyethylenimine system as a potent vaccine to boost the immune response in vivo [32]. The micelles exhibited great antigen-loading capability due to their cationic surface charge and minor cytotoxic effects in vitro. They also significantly improved the OVA antigen uptake by DCs in both in vitro and in vivo experiments. More importantly, the OVA encapsulated in a cationic micellar vaccine could significantly boost the anti-OVA antibody production and significantly improve the T-cell proliferation and the secretion of IL-5 and IFN-γ [32]. The prepared micellar system also exhibited great potential as a vaccine formulation to trigger Th2 immune response [32].

Polymer diblock nanocarriers consisting of an N-(2-hydroxypropyl) methacrylamide corona block with pendent pyridyl disulfide groups for reversible conjugation of thiolated OVA and a terpolymer ampholytic core-forming block composed of propylacrylic acid (PAA), dimethylaminoethyl methacrylate (DMAEMA), and butyl methacrylate (BMA) were synthesized by the authors of [33] and self-assembled into micellar nanoparticles. These nanoparticles had a size of around 30 nm in diameter and were conjugated with OVA. Subcutaneous immunization of mice with these nanoparticulate micellar systems significantly increased antigen-specific CD8(+) T-cell responses (0.4% IFN-γ(+) of CD8(+)) compared to immunization with soluble protein, OVA and polymer mixture, and the control micelle without endosome-releasing activity [33]. These pH-responsive polymeric micelles could find application in vaccine design due to CD8(+) T-cell activation [33].

**Figure 3:**
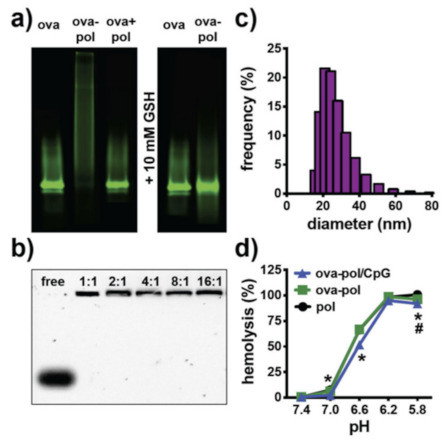
pH-responsive micellar nanocarriers for dual delivery of antigen and oligonucleotides. (**a**) SDS-PAGE of fluorescently labeled ovalbumin (OVA), nanoparticle–OVA conjugates at a polymer/OVA molar ratio of 20:1 (ova-pol), and a physical mixture of ova and polymer (ova+pol). Incubation of conjugates with intracellular concentrations of glutathione (GSH) liberates OVA from the carrier. (**b**) Agarose gel electrophoresis of OVA–nanoparticle conjugates incubated with CpG ODN1826 at various positive/negative charge ratios. (**c**) Representative size distribution (number average) measured by dynamic light scattering (DLS) of ova-pol/CpG complexes at a 4:1 charge ratio. (**d**) Erythrocyte lysis assay demonstrating pH-dependent membrane destabilizing activity of the diblock copolymer micelles (pol), nanoparticle–OVA conjugate (ova-pol), and conjugate complexed with CpG ODN (ova-pol/CpG). Concentrations are normalized to 2.5 μg/mL polymer and data represent mean +/− s.d. (n = 4). * *p* < 0.05: ova−pol/CpG vs. pol and ova−pol; # *p* < 0.05: ova−pol vs. pol by ANOVAwith Tukey’s post hoc test. (Adapted from [34]).

Micellar nanoparticulate systems composed of amphiphilic diblock copolymers (an ampholytic core-forming block and a redesigned polycationic corona block, doped with thiol-reactive pyridyl disulfide groups) were designed and synthesized by Wilson et al. [34]. These micelles were developed for the co-delivery of antigens and immunostimulatory CpG oligodeoxynucleotide (CpG ODN) adjuvants. The size of the micelles was around 25 nm in diameter with the encapsulation of CpG ODN and OVA. Encapsulation of OVA into micelles significantly increased antigen cross-presentation in vitro in comparison to pure OVA or an unformulated physical mixture of the same biomaterials/ingredients of the final formulation. Additionally, the subcutaneous vaccine administration of rats with OVA–polymer micelle complexes induced a significantly higher CD8(+) T-cell response compared to mice administrated with free OVA or the pure unformulated ingredients of the two materials and enhanced CD8(+) T-cell responses relative to immunization with systems, OVA administered with free CpG, or a formulation containing free OVA and CpG complexed to micelles (Figure 3). Similarly, co-delivery carriers significantly increased Th1 responses and elicited a balanced ratio of IgG1 and IgG2c antibody secretion [34]. Transdermal administration further improved the cellular immune responses, with co-delivery systems inducing antigen-specific CD8(+) T cells. This work demonstrated the ability of pH-responsive, endosomatic micelles to actively promote antigen cross-presentation and augment cellular and humoral immune responses via dual-delivery of protein antigens and CpG ODN [34]. For these reasons, pH-responsive polymeric micelles offer numerous advantages as a delivery platform for protein subunit vaccines.

#### 3.1.2. PLGA-Based and Other Biocompatible Nanoparticles

Chitosan-modified, polyethyleneimine-modified, and ε-poly-L-lysine-modified PLGA nanoparticles were prepared as antigen vehicles for the dual delivery of Alhagi honey polysaccharides and OVA [35]. All the prepared modified PLGA systems induced secretion of cytokines, antibodies, and their subtypes (IgG) in immunized mice. These results demonstrate that these formulations generated a strong Th1-biased immune response. Among them, ε-poly-L-lysine-modified PLGA systems induced the strongest Th1-biased immune response [35]. Macrophage immunomodulatory activity of the modified PLGA nanoparticles with cationic surface net charge incorporating Alhagi honey polysaccharide has also been described in the literature [36,37]. PLGA nanoparticles have been used to deliver Angelica sinensis polysaccharides. This system can be developed as a vaccine platform and/or an adjuvant system for OVA [38]. Immunization of mice with the above-described systems could significantly enhance lymphocyte proliferation, improve the ratio of CD4^+^/CD8^+^ T cells, and induce a strong cellular immune response [38]. The same results were detected for Angelica sinensis polysaccharides incorporated with polyethylenimine-coated PLGA nanoparticles [39]. Namely, PLGA nanoparticles and polyethylenimine were used to coat nanosystems in order to develop an innovative nanodelivery vehicle with cationic surface charge net. This formulation activated macrophages and promoted the significant expression of the MHCII and CD86 and the production of IL-1β and IL-12p70 cytokines of macrophages. Furthermore, the antigen anchored on the surface of the ASP-PLGA-PEI improved the antigen uptake by macrophages. Indeed, the immunization of mice with PCV2 antigen-adsorbed ASP-PLGA-PEI nanoparticles significantly enhanced PCV2-specific IgG immune response and the levels of cytokines and induced a mixed Th1/Th2 immune response with Th1 bias compared with other groups [39]. Gu et al. studied how the surface charge and antigen loading of PLGA nanoparticles alter the immune responses [39,40]. The findings demonstrated that PEI-coated (positively charged) nanoparticles boosted the antigen escape from the endosome, which led to the cytoplasmic antigen delivery to generate cross-presentation, compared to nanoparticles with negative surface charge [40]. In addition, PEI-coated nanoparticles activated the DCs in lymph nodes a few days after the system administration. The in vivo experiments showed that the antigen-incorporated nanoparticles induced stronger and long-term antigen-specific antibody responses compared to those of antigen-adsorbed nanoparticles [40].

As mentioned before, antigen-loaded polymer nanoparticles have proven more effective in increasing T-cell responses than the corresponding molecular antigens. The use of hydrophilic PEG-b-PAGE-b-PLGA (PPP) for the preparation of antigen-loaded nanoparticles (NPs) as a platform for prophylactic vaccination has also appeared in the literature [41]. OVA was used as a model antigen, and the authors prepared nanoparticles of different physicochemical characteristics, loading efficiencies, and release kinetics. T-cell activation by antigen-presenting cells was significantly increased in vitro if antigen was delivered via PPP nanosystems compared to PLGA nanoparticles or OVA solution, although antigen content was the same in all tested formulations. Subcutaneous application of PPP-OVA-NPs even without adjuvants led to generation of potent CD8 T-cell-mediated OVA-specific cytotoxicity in vivo that was more pronounced than after application of OVA alone or PLGA-OVA nanosystems.

**Figure 4:**
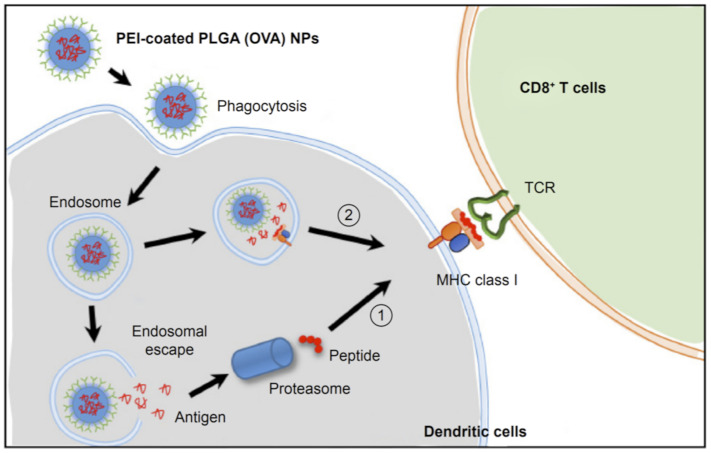
Schematic illustration of the predicted mechanism of cross-presentation and CD8^+^ T-cell response induced by PEI-coated PLGA (OVA) NPs. Notes: OVA encapsulated in PEI-coated PLGA (OVA) NPs entered the DCs through phagocytosis or macropinocytosis, and then (1) OVA escaped and was released from an endosome. The released OVA was processed by a proteasome and presented by MHC class I molecules. (2) OVA was processed by a lysosomal protease in an endosome and cross-presented by MHC class I molecules. Abbreviations: DCs, dendritic cells; PEI, polyethylenimine; PLGA, poly(d,l-lactide-co-glycolide); OVA, ovalbumin; NPs, nanoparticles; MHC, major histocompatibility complex; TCR, T-cell receptor. (Adapted from [42]).

The PEI-coated PLGA (OVA) nanoparticles can induce antigen cross-presentation and are expected to be used for induction of a strong cytotoxic T-lymphocyte immune response and for efficient anticancer immunotherapy, according to Song et al. [42]. PEI-coated PLGA OVA nanoparticles were internalized efficiently via phagocytosis or macropinocytosis in DCs and induced efficient cross-presentation of the antigen on MHC class I molecules via both endosome escape and a lysosomal processing mechanism. The dendritic cells treated with PEI-coated PLGA (OVA) nanoparticles induced a release of IL-2 cytokine from OVA-specific CD8-OVA1.3 T cells more efficiently than dendritic cells treated with PLGA (OVA) nanoparticles. A schematic illustration of the predicted mechanism of cross-presentation and CD8^+^ T-cell response induced by PEI-coated PLGA (OVA) is presented in Figure 4.

According to Koerner et al., improved vaccine effectiveness of the polymer-based systems is attributed to controlled release of incorporated antigens, specific targeting of APCs, and subsequent induction of cytotoxic T-lymphocyte immunity [43]. PLGA, as one of these polymers, has been extensively studied for the design and development of particulate antigen delivery systems in cancer therapy [43].

The loading and the delivery of antigens with poly(propylacrylic acid) complexation improves both MHC-1 presentation and T-cell activation [44]. These polymeric nanovectors enhancing cytosolic delivery of antigens with protein nature could lead to high CD8^+^ T-cell response and demonstrate the capability of pH-responsive PPAA-based systems for therapeutic vaccine usage [45].

Chitosan-based nanoparticles were developed for improving immunization against hepatitis B infection [46]. Polysaccharide-based nanoparticles were prepared using a very mild ionic gelation technique by cross-linking the polysaccharide chitosan (CS) with a counter ion and using recombinant hepatitis B surface antigen (rHBsAg) as a model [46]. The in vivo experiments showed that this system is a promising adjuvant for vaccine delivery of subunit antigens because it induced anti-HBsAg IgG levels up to 5500 mIU/mL, values 9-fold higher than the conventional alum-adsorbed vaccine [46]. Alginate-coated chitosan nanoparticles are also an effective subcutaneous adjuvant for hepatitis B surface antigen [47].

Surface coated poly-(ɛ-caprolactone) (PCL) nanoparticles with chitosan (CS) were developed as a carrier system for nasal immunization using recombinant influenza A virus (A/California/07/2009) H1N1 hemagglutinin (HA) protein for the induction of humoral, cellular, and mucosal immunity [48]. The nanoparticles induced balanced Th1 and Th2 responses and produced humoral (both systemic and mucosal) and cellular immune responses upon nasal administration [48].

Matsuo et al. developed a hydrogel patch formulation to promote the penetration of antigenic proteins into the stratum corneum [49,50]. The hydrogel patch formulation, comprising cross-linked acrylate medical adhesives octyldodecyl lactate/glycerin/sodium hyaluronan = 100:45:30:0.2 as weight ratio of composition, was prepared. The transcutaneous immunization system induced toxoid-specific IgG production in an antigen dose-, patch area-, and application period-dependent manner for tetanus and diphtheria [49,50].

Microcapsules of biodegradable polyelectrolytes dextran sulfate (DEXS) and poly-L-arginine (pARG) were formulated by layer-by-layer technology, and PLGA microparticles were prepared by spray-drying [51]. All the systems were loaded with model antigen OVA [51]. Mice were immunized by subcutaneous administration either by a single injection or by two injections separated by one month with an equivalent dose of the OVA-encapsulated platforms. Both platforms mediated high, long-term IgG(1) responses, whereas the IgG(2c) titers remained low. Additionally, Th1 and Th2 phenotype immune responses against OVA were assessed by quantifying the production of cytokines in CD4^+^ T cells derived from the spleens of immunized mice at 6 months after the first injection. Immunization with systems led to significantly increased IL-2, IL-4, IL-10, and IFN-γ production by splenic CD4^+^ T cells compared to control animals [51]. Layer-by-layer microcapsules and PLGA microparticles generated strong immune responses in vivo, characterized by a Th1/Th2 type response with predominance of Th2 immunity. Both particulate formulations elicited a comparable type of immune response and appear to be promising for antigen delivery [51]. Co-encapsulation of OVA and CpG oligonucleotides into PLGA microparticles has been also achieved [52]. The authors prepared 1-micron near non-charged PLGA 502 and PLGA 756 microparticles that were loaded with 50% (encapsulation efficiency) ovalbumin (OVA), approximately, into their matrix and CpG-chitosan complexes (near 20%) onto their surface, maintaining the integrity of OVA and CpG. In the intradermal immunization studies, OVA microencapsulated into PLGA 756 alone induced a strong humoral immune response assisted by a very clear Th1 bias that was decreased by CpG co-delivery (IgG2a/IgG1 = 0.55). The co-encapsulation of CpG with OVA in PLGA 502 particles significantly improved the antibody response and isotype shifting in comparison with mice immunized with OVA-loaded PLGA 502. These results showed the crucial and central role of polymer nature and the physicochemical characteristics of particles to prove the benefits of co-incorporating CpG motifs in close proximity with OVA [52].

It is well known that oral vaccination has several advantages over the commonly used and marketed products with parenteral routes of administration. On the other hand, the decomposition of oral vaccine formulations and their limited uptake in the lymphoid tissue of the gastrointestinal (GI) tract still act as a gap for their further scale-up. Sarti et al. utilized the OVA as model antigen and monophosphoryl lipid A (MPLA), a lipid with immunostimulant properties, and they incorporated them into polymeric nanoparticles composed of PLGA [53]. The prepared nanosystems were orally administered to Bagg albino mice. The results, which showed time-controlled immune responses (both systemic and mucosal) towards OVA, were assessed by quantifying the IgG and IgA levels, which were OVA-specific using ELISA. PLGA nanoparticulate systems were spherical in morphology, around 320 nm in size (diameter), negatively surface charged (zeta potential around −20 mV), and had OVA and MPLA encapsulation efficiencies of 9.6% and 0.86%, respectively. A single-dose immunization with a formulation containing both OVA and MPLA incorporated in PLGA nanoparticles induced a very strong IgG immune response in comparison to those induced by OVA in PBS dispersion and OVA incorporated into PLGA nanoparticles. Moreover, significantly higher IgA levels were observed by administration of OVA and MPLA PLGA nanoparticles compared to IgA stimulated by pure formulations, proving the ability to enhance the mucosal immune response [53]. These findings demonstrate that dual delivery of OVA and MPLA in PLGA nanoparticles promotes both systemic and mucosal immune responses and, therefore, represents a suitable strategy for oral vaccination [53].

On the other hand, nasal vaccination is a promising, needle-free alternative to classical vaccination. Slutter et al. [54] correlated the differences in physicochemical characteristics of nanoparticles to their adjuvant effect, using OVA-loaded PLGA nanoparticles, N-trimethyl chitosan (TMC)-based nanoparticles, and TMC-coated PLGA nanoparticles. PLGA and PLGA/TMC nanoparticles were prepared by emulsification/solvent extraction, and TMC nanoparticles were prepared by ionic complexation method. The TMC nanoparticles increased the nasal residence time of OVA compared to OVA administered in solution and induced DC maturation. After intramuscular administration, all the nanoparticulate delivery platforms induced higher IgG levels than pure OVA; PLGA and TMC nanoparticles were more effective in comparison to PLGA TMC nanoparticles. Nasal immunization with the slow antigen release profile, PLGA, and PLGA/TMC nanoparticles did not produce detectable antibody levels. On the contrary, nasal administration with the positively charged, fast TMC nanoparticles showed high serum antibody and sIgA levels probably due to fast antigen release. In conclusion, particle charge and antigen release profile of OVA-loaded nanoparticles must be adapted to the intended route of administration. For nasal vaccination, TMC nanoparticles, with long time (hours) of OVA release, mucoadhesive nature, and ability to stimulate the maturation of DCs, were more effective in comparison to PLGA nanoparticles and PLGA/TMC nanoparticles, which were characterized by different physicochemical, morphological, and release profile characteristics [54].

The outer membrane protein antigen of Aeromonas hydrophila was incorporated in PLA and PLGA nanoparticulate systems [55]. The immunogenicity of the prepared nanoparticles was evaluated through intraperitoneal injection in fish, Labeo rohita [55]. The incorporation efficiency of the antigen was quite low, but the specific antibody response was significantly increased and persisted up over a month after immunization by both developed nanoformulation vehicles [55]. According to these results, both the PLA and PLGA nanoparticles could be novel antigen carriers for parenteral immunization in fish [55].

**Figure 5:**
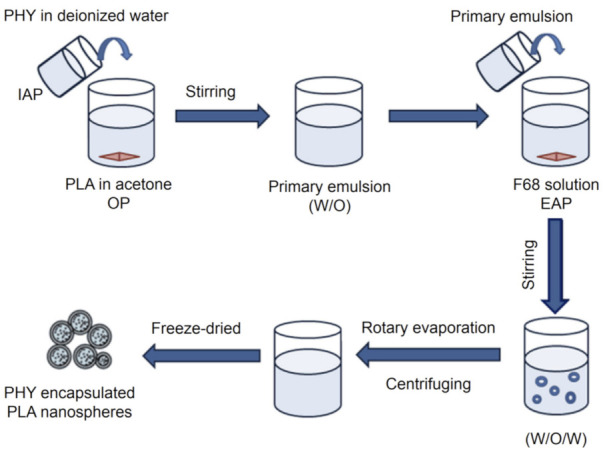
Schematic illustration of the fabrication process of PHYP and empty PLA nanospheres. Note: The formulations were prepared by the double emulsion solvent evaporation method and stored at 4 °C prior to usage. Abbreviations: PHYP, pachyman-loaded poly(d,l-lactic acid); PLA, polylactide; PHY, pachyman; IAP, internal aqueous phase; OP, organic phase; EAP, external aqueous phase; w/o, water in oil; w/o/w, water in oil in water. (Adapted from [56]).

Maturation of DCs in vitro and immunological enhancement of mice in vivo by pachyman- and/or OVA-encapsulated PLA nanospheres was reported in recent literature [56,57]. A schematic illustration of the fabrication process of pachyman-loaded and empty PLA nanospheres is presented in Figure 5. Namely, the results showed that, when stimulated by pachyman, the bone marrow DCs matured because of upregulated expression of co-stimulatory substances. The mice inoculated with OVA–pachyman had augmented IgG antibodies, increased cytokine secretion by splenocytes, increased splenocyte proliferation, and activation of cluster of differentiation (CD)4^+^ and CD8^+^ T cells in vivo. Elevated immune responses were produced by OVA–pachyman, possibly owing to the activation and maturation of dendritic cells (in draining lymph nodes). Furthermore, surface modification of PLGA nanoparticles with protamine enhanced the cross-presentation of encapsulated OVA by bone marrow-derived DCs [58]. The results showed that the cross-presentation of encapsulated exogenous antigen was increased by improving antigen uptake and lysosomal escape, suggesting the feasibility to be a potent adjuvant vaccine [58].

Additionally, targeting dendritic cells with nanoparticulate PLGA cancer vaccine formulations is a new trend in the literature [59,60,61]. Mouse immunization with pH-responsive PLGA nanoparticles induced greater lymphocyte activation, more antigen-specific CD8(+) T cells, stronger cytotoxic capacity (IFN-γ and granzyme B), enhanced antigen-specific IgG antibodies, and higher serum IgG2a/IgG1, indicating cellular immunity [59,60,61].

### 3.2. The Added Value of Polymer-Based Nanovaccines

We presented several examples from the recent literature about polymer-based nanovaccines and in some cases polymer microparticulate vaccine platforms. Generally, different types of nanoparticles have been already used as antigen vehicles and/or particulate adjuvant in the field of novel vaccines. As mentioned before, Table 1 summarizes the different types of nanoparticles, their characteristics, and their disadvantages. Additionally, we presented several polymers with different architectures used as vaccine platforms in the previous section, but a high percentage of the published studies deal with PLGA. Table 2 summarizes the main advantages and disadvantages of PLGA-based particulate vaccine delivery systems.

The polymer-based nanovaccines exhibit several advantages:

1. Strong cellular immune responses [38,42];
2. Increased secretion of cytokines [58];
3. Different routes of administration [47,49,50,53];
4. Co-loading of antigens [34,52];
5. Prolonged antigen circulation [43];
6. Increased levels of antibodies and antigen-specific antibodies (i.e., IgA, IgG, etc.) [46,47,58];
7. Th1 and/or Th2 immune responses [40,51];
8. Advanced adjuvant properties [38];
9. Single-dose formulations [47,49,50,53];
10. Needle-free dosage forms [47,49,50,53].

Other nanoparticles, e.g., liposomes, exhibit some of these advantages [66,67,68,69], but only polymer-based nanovaccines can present all the above advantages. Furthermore, comparative studies of biodegradable nanoparticles composed of poly(glutamic acid) nanoparticles with aluminum adjuvants, which are already used in commercial vaccines, showed an excellent antigen uptake by dendritic cells (localized in the lysosomal regions) and adjuvant activity, as well as induction of immune response in mice via a TLR4 and MyD88 signaling pathway [70,71]. These biodegradable nanoparticles are effective for carrying different types of antigens and also exhibit antigen-specific humoral and cellular immunity [70,71,72,73,74]. The cellular and the humoral responses are strongly dependent on the architecture and the chemistry of the hydrophobic polymer chains and the formulation protocol of the nanoparticulate vaccine platform (encapsulation or mixture) [75,76]. The size of these nanoparticles plays a significant role in the uptake and activation behaviors of APCs migrating to lymph nodes and DC maturation [77]. Namely, the sizes above 100 nm are ideal for cellular uptake, while the sizes below 100 nm are perfect for the maturation of DCs in lymph nodes [77]. The surface coatings and/or surface decorations of nanogels influence the interferon-γ production by T lymphocytes [78]. The presence of PEG as surface coating and the length of its chain influence the antibody–receptor interactions and induction of antigen-specific T-cell responses [79]. Furthermore, the immunization with polymer nanoparticles induces high antibody rates compared to other nanosystems such as liposomes and alum [69,70,71,72,73,74,75,76,77,78,79,80,81,82,83,84,85].

**Figure 6:**
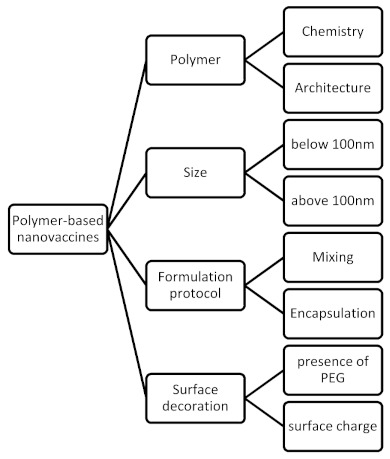
The formulation parameters of the design and development of polymer-based nanovaccines.

As mentioned above, from the technological point of view, polymers and, consequently, polymer-based nanoparticles offer design versatility. Firstly, there are different types (compositions and architectures) of polymers. Different types of nanoparticles can be prepared due to different types of polymers (i.e., micelles, polymersomes, hydrogels, polymeric nanoparticles, hybrid particles, etc.). The physicochemical characteristics of these nanosystems are crucial for their behavior in vitro and in vivo. The surface hydrophobicity and size are the most important formulation parameters for the creation of antigen-specific antibodies [85]. The formulation parameters of the design and development of polymer-based nanovaccines are presented in Figure 6. Additionally, the lower cost of polymers in comparison to other materials, i.e., lipids, dendrimers, etc., and their large-scale and commercial use make polymer-based nanovaccines attractive for the pharmaceutical companies from the development point of view.

### 3.3. The Limitations in the Development of Polymer-Based Nanovaccines

We have described several examples of polymer-based nanovaccines that have appeared in the literature in different stages of preclinical and clinical studies. The results and the outcomes were in most of the cited cases very optimistic for further development of polymer-based platforms for vaccine applications. On the other hand, the number of polymer-based nanovaccines is close to zero, while there are several polymer nanomedicines on the market [84,85,86,87]. Firstly, to push forward the application of polymer-based nanovaccines, it would be important that more groups from different fields (from synthetic chemists and formulation scientists to clinical doctors and regulatory scientists) be actively involved in the testing of new vaccine platforms against different infectious diseases. This collaboration is the first and important step to overcome the potential shortcomings of polymer-based nanovaccines. Other important issues deserving attention include the following: (a) The synthesis of new polymers with low nanotoxicity and low immunogenicity is one of the limitations of the usage of new polymers in the further development of polymer therapeutics [84,85,86,87]. (b) The in-depth investigation of the physicochemical and morphological properties of the prepared polymer formulations, by using specialized techniques, is mandatory for all the nanomedicines under development. This need increases the cost of the preclinical studies in comparison to other pharmaceutical formulations [84,85,86,87]. (c) The pharmaceutical industries should change or further develop the instrumentation for the production and quality control of the polymer-based nanovaccines. Scientists with qualification in the field of polymers are also required in big pharma at every stage of the design and the development of polymer-based nanovaccines [84,85,86,87]. Last but not least, the regulatory landscape is not very clear containing grey areas for the nanoformulations, and this is an additional difficulty for the preparation of the dossier of a new vaccine. All these limitations act as a brake towards the acceleration of the development of polymer-based nanovaccines [84,85,86,87,88,89,90]. However, all these limitations could be a challenge and an opportunity for the strongest collaboration of scientists to overcome them.

## 4. Conclusions and Future Perspectives

Polymer-based systems are very well known drug delivery carriers in pharmaceutical nanotechnology and nanomedicine. Polymer-based nanomedicines are available as market pharmaceutical products with numerous advantages over the conventional formulations. The low toxicity and the biodegradability of most of the polymers used in formulation science make them ideal candidates for the delivery of several therapeutic compounds. The attractive and unique properties of polymer-based materials also make them ideal delivery platforms for antigens. A new avenue in vaccinology was opened by the introduction of polymers in this area of research. The polymer-based nanovaccines are also under intense investigation for the pandemic of coronavirus disease 2019 (COVID-19) [90,91,92,93]. Polymer materials can be used as delivery vehicles in various forms and morphologies for mRNA, DNA, and/or adjuvants in vaccines [90,91,92,93].

This review focuses on polymer-based nanovaccines. Several examples, the properties, the characteristics, the added value, and the limitations of the polymer-based nanovaccines, as well as the process of their development by the pharmaceutical industry, are analyzed in this review. The polymer-based nanovaccines exhibit several advantages such as strong cellular immune responses, increased secretion of cytokines, co-loading and prolonged circulation of antigens, and increased levels of antibodies and antigen-specific antibodies (i.e., IgA, IgG, etc.). Additionally, different and needle-free routes of administration are available for the polymer-based nanovaccines. Finally, polymer nanomaterials exhibit adjuvant properties. Considering the formulation diversity of polymer materials, polymer-based nanovaccines are a new horizon in immunology and vaccinology in the coming years.

## Figures and Tables

**Table 1:** Characteristics of different types of nanoparticles used in vaccines. (Adapted from [65]).

Types	Size	Shape	Bio-Toxicity	Biocompatibility	Disadvantages
VPLs	20–800 nm	Particle	Some unpredictable consequences	Highly cross-protective antibody responses	Low immunogenicity
SAP	10 nm length (70 nm) and wide (40 nm)	Spherical or barrel	-	Reduce the risk of immunogenicity; improve half-life	Creates a new molecular entity; poor solution stability and aggregation
CNPs	30–60 nm length (100–1000 nm) and diameter (0.8–2.0 nm)	Fullerene particle, Nanotube, or mesoporous spheres	Negligible effect on cell viability	Good biocompatibility	Low immune function
GNs	2–150 nm	Particle, rod, spherical, and cubic	Immuno-toxicity	More potent immune response	Immuno-toxicity
CNs	50–100 nm	Calcium phosphate particle	Biocompatible and safe	Biocompatibility and easily biodegradable	-
SNs	50–20 nm	Tunable hollow and mesoporous structure	Toxicity degraded	Biocompatibility and selective tumor targeting, real-time multimodal imaging, vaccine delivery	Toxicity derived from the reducing agents
LSs	25–1000 nm	Spherical	Safely degraded	Stabilize the antigen, biocompatible and stable	-
Polymer	10–2000 nm	Particles	Non-toxicity	Antigen loading into polymeric particles under aqueous conditions via a self-healing process	Loss of antigenicity and immunogenicity during particle synthesis
ISCOM	40 nm	Cage-like particles	Cytotoxicity-mediated immune responses	-	Cytotoxicity-mediated immune responses
EN	50–600 nm	Cytotoxicity-mediated immune responses	Safe	Safe and potent vaccine adjuvant	-

VLPs, virus-like particles; SAP, self-assembled protein; CNPs, carbon nanoparticles; GNs, gold nanomaterials; CNs, calcium nanoparticles; SNs, silica nanoparticles; ISCOM, immunostimulating complex; LSs, liposomes; EN, emulsion.

**Table 2:** Summary of the main advantages and disadvantages of PLGA-based particulate vaccine delivery systems. (Adapted from [66]).

Advantages	Disadvantages
PLGA polymers are biodegradable, widely available, and approved by regulatory agencies such as the FDA PLGA particles for delivery of several different agents are on the market PLGA particles can be administered via various routes PLGA particles may decrease toxicity of vaccine components Particle size, surface, and/or release characteristics can be tailored PLGA particles allow controlled antigen release PLGA particles protect antigen from degradation and elimination PLGA particles enhance antigen uptake by APCs by mimicking size and shape of pathogens PLGA particles enhance and prolong antigen cross-presentation efficiency PLGA particles allow concomitant delivery of multiple vaccine components Large surface area and surface functional groups allow conjugating of targeting moieties PLGA particles may lead to antigen dose sparing	Negative charge of PLGA particles is disadvantageous for particle uptake PLGA particle preparation process must be tailored to the properties of the antigen PLGA particles cannot be sterile filtered Antigen degradation may occur during preparation, storage, and release Antigen release is often incomplete Particle aggregation may occur Particle size may limit crossing of biological barriers

## Data Availability

Not applicable.

## References

[1] Q&As on COVID-19 and Related Health Topics. https://www.who.int/vaccines/questions-and-answers/q-a-on-vaccines.

[2] Zhang Y., Lin S., Wang X.-Y., Zhu G. (2019). Nanovaccines for cancer immunotherapy. Wiley Interdiscip. Rev. Nanomed. Nanobiotechnol..

[3] Bernocchi B., Carpentier R., Betbeder D. (2017). Nasal nanovaccines. Int. J. Pharm..

[4] Vijayan V., Mohapatra A., Uthaman S., Park I.-K. (2019). Recent Advances in Nanovaccines Using Biomimetic Immunomodulatory Materials. Pharmaceutics.

[5] Gregory A.E., Titball R., Williamson D. (2013). Vaccine delivery using nanoparticles. Front. Cell. Infect. Microbiol..

[6] Bhardwaj P., Bhatia E., Sharma S., Ahamad N., Banerjee R. (2020). Advancements in prophylactic and therapeutic nanovaccines. Acta Biomater..

[7] Gregory A.E., Titball R., Williamson D., Reichmuth A.M., Oberli M.A., Jaklenec A., Langer R., Blankschtein D. (2016). mRNA vaccine delivery using lipid nanoparticles. Ther. Deliv..

[8] Kheirollahpour M., Mehrabi M., Dounighi N.M., Mohammadi M., Masoudi A. (2020). Nanoparticles and Vaccine Development. Pharm. Nanotechnol..

[9] Kasturi S.P., Kozlowski P.A., Nakaya H.I., Burger M.C., Russo P., Pham M., Kovalenkov Y., Silveira E.L.V., Haven-ar-Daughton C., Burton S.L. (2017). Adjuvating a Simian Immunodeficiency virus vaccine with Toll-Like Receptor Ligands Encapsulated in Nanoparticles induces Persistent Antibody responses and enhanced protection in TRIM5a restrictive Macaques. J. Virol..

[10] Campos E.V.R., Pereira A.E.S., de Oliveira J.L., Carvalho L.B., Guilger-Casagrande M., de Lima R., Fraceto L.F. (2020). How can nanotechnology help to combat COVID-19? Opportunities and urgent need. J. Nanobiotechnol..

[11] Chakravarty M., Vora A. (2021). Nanotechnology-based antiviral therapeutics. Drug Deliv. Transl. Res..

[12] Yun C.-H., Cho C.-S. (2020). Nanoparticles to Improve the Efficacy of Vaccines. Pharmaceutics.

[13] Dykman L.A. (2020). Gold nanoparticles for preparation of antibodies and vaccines against infectious diseases. Expert Rev. Vaccines.

[14] Liu Y., Guo J., Huang L. (2020). Modulation of tumor microenvironment for immunotherapy: Focus on nanomaterial-based strategies. Theranostics.

[15] Thomas C., Rawat A., Hope-Weeks L., Ahsan F. (2011). Aerosolized PLA and PLGA Nanoparticles Enhance Humoral, Mucosal and Cytokine Responses to Hepatitis B Vaccine. Mol. Pharm..

[16] Diwan M., Tafaghodi M., Samuel J. (2002). Enhancement of immune responses by co-delivery of a CpG oligodeoxynucleotide and tetanus toxoid in biodegradable nanospheres. J. Control. Release.

[17] Manish M., Rahi A., Kaur M., Bhatnagar R., Singh S. (2013). A Single-Dose PLGA Encapsulated Protective Antigen Domain 4 Nanoformulation Protects Mice against Bacillus anthracis Spore Challenge. PLoS ONE.

[18] Lima V.M., Bonato V.L., Lima K.M., Dos Santos S.A., Dos Santos R.R., Goncalves E.D., Faccioli L.H., Brandao I.T., Ro-drigues-Junior J.M., Silva C.L. (2001). Role of trehalose dimycolate in recruitment of cells and modulation of protection of cytokines and NO in tuberculosis. Infect. Immun..

[19] Borges O., Cordeiro-Da-Silva A., Tavares J., Santarém N., de Sousa A., Borchard G., Junginger H.E. (2008). Immune response by nasal delivery of hepatitis B surface antigen and codelivery of a CpG ODN in alginate coated chitosan nanoparticles. Eur. J. Pharm. Biopharm..

[20] Li P., Luo Z., Liu P., Gao N., Zhang Y., Pan H., Liu L., Wang C., Cai L., Ma Y. (2013). Bioreducible alginate-poly(ethylenimine) nanogels as an antigen delivery sytems robustly enhance vaccine-elicited humoral and cellular immune responses. J. Control. Release.

[21] Hasegawa K., Noguchi Y., Koizumi F., Uenaka A., Tanaka M., Shimono M., Nakamura H., Shiku H., Gnjatic S., Murphy R. (2006). In vitro Stimulation of CD8 and CD4 T Cells by Dendritic Cells Loaded with a Complex of Cholesterol-Bearing Hydrophobized Pullulan and NY-ESO-1 Protein: Identification of a New HLA-DR15–Binding CD4 T-Cell Epitope. Clin. Cancer Res..

[22] Saade F., Honda-Okubo Y., Trec S., Petrovsky N. (2013). A novel hepatitis B vaccine containing Advax™, a polysaccharide adjuvant derived from delta inulin, induces robust humoral and cellular immunity with minimal reactogenicity in preclinical testing. Vaccine.

[23] Feng G., Jiang Q., Xia M., Lu Y., Qiu W., Zhao D., Lu L., Peng G., Wang Y. (2013). Enhanced Immune Response and Protective Effects of Nano-chitosan-based DNA Vaccine Encoding T Cell Epitopes of Esat-6 and FL against Mycobacterium Tuberculosis Infection. PLoS ONE.

[24] Glaffig M., Palitzsch B., Hartmann S., Schüll C., Nuhn L., Gerlitzki B., Schmitt E., Frey H., Kunz H. (2014). A Fully Synthetic Glycopeptide Antitumor Vaccine Based on Multiple Antigen Presentation on a Hyperbranched Polymer. Chem. A Eur. J..

[25] Luo Z., Li P., Deng J., Gao N., Zhang Y., Pan H., Liu L., Wang C., Cai L., Ma Y. (2013). Cationic polypeptide micelle-based antigen delivery system: A simple and robust adjuvant to improve vaccine efficacy. J. Control. Release.

[26] Zhang C., Shi G., Zhang J., Song H., Niu J., Shi S., Huang P., Wang Y., Wang W., Li C. (2017). Targeted antigen delivery to dendritic cell via functionalized alginate nanoparticles for cancer immunotherapy. J. Control. Release.

[27] Yan Y., Ding H. (2020). pH-Responsive Nanoparticles for Cancer Immunotherapy: A Brief Review. Nanomaterials.

[28] Démoulins T., Bassi I., Thomann-Harwood L., Jandus C., Kaeuper P., Simon H.-U., von Gunten S., McCullough K.C. (2013). Alginate-coated chitosan nanogel capacity to modulate the effect of TLR ligands on blood dendritic cells. Nanomed. Nanotechnol. Biol. Med..

[29] Démoulins T., Milona P., McCullough K.C. (2014). Alginate-coated chitosan nanogels differentially modulate class-A and class-B CpG-ODN targeting of dendritic cells and intracellular delivery. Nanomedicine.

[30] Trimaille T., Verrier B. (2015). Micelle-Based Adjuvants for Subunit Vaccine Delivery. Vaccines.

[31] Trimaille T., Lacroix C., Verrier B. (2019). Self-assembled amphiphilic copolymers as dual delivery system for immunotherapy. Eur. J. Pharm. Biopharm..

[32] Luo Z., Shi S., Jin L., Xu L., Yu J., Chen H., Li X. (2015). Cationic micelle based vaccine induced potent humoral immune response through enhancing antigen uptake and formation of germinal center. Colloids Surf. B Biointerfaces.

[33] Keller S., Wilson J.T., Patilea G.I., Kern H.B., Convertine A.J., Stayton P.S. (2014). Neutral polymer micelle carriers with pH-responsive, endosome-releasing activity modulate antigen trafficking to enhance CD8(+) T cell responses. J. Control. Release.

[34] Wilson J.T., Keller S., Manganiello M.J., Cheng C., Lee C.C., Opara C., Convertine A., Stayton P.S. (2013). pH-Responsive nanoparticle vaccines for dual-delivery of antigens and immunostimulatory oligonucleotides. ACS Nano.

[35] Wusiman A., Gu P., Liu Z., Xu S., Zhang Y., Hu Y., Liu J., Wang D., Huang X. (2019). Cationic polymer modified PLGA nano-particles encapsulating Alhagi honey polysaccharides as a vaccine delivery system for ovalbumin to improve immune responses. Int. J. Nanomed..

[36] Wusiman A., He J., Zhu T., Liu Z., Gu P., Hu Y., Liu J., Wang D. (2019). Macrophage immunomodulatory activity of the cati-onic polymer modified PLGA nanoparticles encapsulating Alhagi honey polysaccharide. Int. J. Biol. Macromol..

[37] Wusiman A., Xu S., Ni H., Gu P., Liu Z., Zhang Y., Qiu T., Hu Y., Liu J., Wu Y. (2019). Immunomodulatory effects of Alhagi honey polysaccharides encapsulated into PLGA nanoparticles. Carbohydr. Polym..

[38] Gu P., Liu Z., Sun Y., Ou N., Hu Y., Liu J., Wu Y., Wang D. (2019). Angelica sinensis polysaccharide encapsulated into PLGA nanoparticles as a vaccine delivery and adjuvant system for ovalbumin to promote immune responses. Int. J. Pharm..

[39] Gu P., Wusiman A., Wang S., Zhang Y., Liu Z., Hu Y., Liu J., Wang D. (2019). Polyethylenimine-coated PLGA nanoparticles-encapsulated Angelica sinensis polysaccharide as an adjuvant to enhance immune responses. Carbohydr. Polym..

[40] Gu P., Wusiman A., Zhang Y., Liu Z., Bo R., Hu Y., Liu J., Wang D. (2019). Rational Design of PLGA Nanoparticle Vaccine Delivery Systems To Improve Immune Responses. Mol. Pharm..

[41] Rietscher R., Schröder M., Janke J., Czaplewska J., Gottschaldt M., Scherließ R., Hanefeld A., Schubert U.S., Schneider M., Knolle P.A. (2016). Antigen delivery via hydrophilic PEG-b-PAGE-b-PLGA nanoparticles boosts vac-cination induced T cell immunity. Eur. J. Pharm. Biopharm..

[42] Lim Y.T., Song C., Noh Y.-W. (2016). Polymer nanoparticles for cross-presentation of exogenous antigens and enhanced cytotoxic T-lymphocyte immune response. Int. J. Nanomed..

[43] Koerner J., Horvath D., Groettrup M. (2019). Harnessing Dendritic Cells for Poly (D,L-lactide-co-glycolide) Microspheres (PLGA MS)—Mediated Anti-tumor Therapy. Front. Immunol..

[44] Flanary S., Hoffman A.S., Stayton P.S. (2009). Antigen Delivery with Poly(Propylacrylic Acid) Conjugation Enhances MHC-1 Presentation and T-Cell Activation. Bioconjug. Chem..

[45] Foster S., Duvall C.L., Crownover E.F., Hoffman A.S., Stayton P.S. (2010). Intracellular delivery of a protein antigen with an en-dosomal-releasing polymer enhances CD8 T-cell production and prophylactic vaccine efficacy. Bioconjug. Chem..

[46] Prego C., Paolicelli P., Díaz B., Vicente S., Sánchez A., González-Fernández Á., Alonso M.J. (2010). Chitosan-based nanoparticles for improving immunization against hepatitis B infection. Vaccine.

[47] Borges O., Silva M., de Sousa A., Borchard G., Junginger H.E., Cordeiro-da-Silva A. (2008). Alginate coated chitosan nanoparticles are an effective subcutaneous adjuvant for hepatitis B surface antigen. Int. Immunopharmacol..

[48] Gupta N.K., Tomar P., Sharma V., Dixit V.K. (2011). Development and characterization of chitosan coated poly-(ɛ-caprolactone) nanoparticulate system for effective immunization against influenza. Vaccine.

[49] Matsuo K., Ishii Y., Quan Y.S., Kamiyama F., Mukai Y., Yoshioka Y., Okada N., Nakagawa S. (2011). Transcutaneous vaccina-tion using a hydrogel patch induces effective immune responses to tetanus and diphtheria toxoid in hairless rat. J. Control. Release.

[50] Hirobe S., Matsuo K., Quan Y.-S., Kamiyama F., Morito H., Asada H., Takaya Y., Mukai Y., Okada N., Nakagawa S. (2012). Clinical study of transcutaneous vaccination using a hydrogel patch for tetanus and diphtheria. Vaccine.

[51] De Temmerman M.-L., Rejman J., Vandenbroucke R., De Koker S., Libert C., Grooten J., Demeester J., Gander B., De Smedt S.C. (2012). Polyelectrolyte LbL microcapsules versus PLGA microparticles for immunization with a protein antigen. J. Control. Release.

[52] San Román B., Irache J.M., Gómez S., Tsapis N., Gamazo C., Espuelas M.S. (2008). Co-encapsulation of an antigen and CpG oligonucleotides into PLGA microparticles by TROMS technology. Eur. J. Pharm. Biopharm..

[53] Sarti F., Perera G., Hintzen F., Kotti K., Karageorgiou V., Kammona O., Kiparissides C., Bernkop-Schnürch A. (2011). In vivo evidence of oral vaccination with PLGA nanoparticles containing the immunostimulant monophosphoryl lipid A. Biomaterials.

[54] Slütter B., Bal S., Keijzer C., Mallants R., Hagenaars N., Que I., Kaijzel E., van Eden W., Augustijns P., Löwik C. (2010). Nasal vaccination with N-trimethyl chitosan and PLGA based nanoparticles: Nanoparticle charac-teristics determine quality and strength of the antibody response in mice against the encapsulated antigen. Vaccine.

[55] Rauta P.R., Nayak B. (2015). Parenteral immunization of PLA/PLGA nanoparticle encapsulating outer membrane protein (Omp) from Aeromonas hydrophila: Evaluation of immunostimulatory action in Labeo rohita (rohu). Fish Shellfish Immunol..

[56] Wang D., Zheng S., Luo L., Bo R., Liu Z., Xing J., Niu Y., Hu Y., Liu J. (2016). Evaluation of optimum conditions for pachyman encapsulated in poly(D,L-lactic acid) nanospheres by response surface methodology and results of a related in vitro study. Int. J. Nanomed..

[57] Zheng S., Qin T., Lu Y., Huang Y., Luo L., Liu Z., Bo R., Hu Y., Liu J., Wang D. (2018). Maturation of dendritic cells in vitro and immunological enhancement of mice in vivo by pachyman- and/or OVA-encapsulated poly(D,L-lactic acid) nanospheres. Int. J. Nanomed..

[58] Han R., Zhu J., Yang X., Xu H. (2011). Surface modification of poly(D,L-lactic-co-glycolic acid) nanoparticles with protamine enhanced cross-presentation of encapsulated ovalbumin by bone marrow-derived dendritic cells. J. Biomed. Mater. Res. A.

[59] Liu Q., Chen X., Jia J., Zhang W., Yang T., Wang L., Ma G. (2015). pH-Responsive Poly(d,l-lactic-co-glycolic acid) Nanoparticles with Rapid Antigen Release Behavior Promote Immune Response. ACS Nano.

[60] Heo M.B., Cho M.Y., Lim Y.T. (2014). Polymer nanoparticles for enhanced immune response: Combined delivery of tumor antigen and small interference RNA for immunosuppressive gene to dendritic cells. Acta Biomater..

[61] Hamdy S., Haddadi A., Hung R.W., Lavasanifar A. (2011). Targeting dendritic cells with nano-particulate PLGA cancer vaccine formulations. Adv. Drug Deliv. Rev..

[62] Li L., Yang Z., Chen X. (2020). Recent Advances in Stimuli-Responsive Platforms for Cancer Immunotherapy. Acc. Chem. Res..

[63] Aikins M.E., Xu C., Moon J.J. (2020). Engineered Nanoparticles for Cancer Vaccination and Immunotherapy. Acc. Chem. Res..

[64] Xia Y., Song T., Hu Y., Ma G. (2020). Synthetic Particles for Cancer Vaccines: Connecting the Inherent Supply Chain. Acc. Chem. Res..

[65] Shen Y., Hao T., Ou S., Hu C., Chen L. (2017). Applications and perspectives of nanomaterials in novel vaccine development. MedChemComm.

[66] Silva A.L., Soema P.C., Slütter B., Ossendorp F., Jiskoot W. (2016). PLGA particulate delivery systems for subunit vaccines: Linking particle properties to immunogenicity. Hum. Vaccines Immunother..

[67] Jiang W., Gupta R.K., Deshpande M.C., Schwendeman S.P. (2005). Biodegradable poly(lactic-co-glycolic acid) microparticles for injectable delivery of vaccine antigens. Adv. Drug Deliv. Rev..

[68] Sah H., Thoma L.A., Desu H.R., Sah E., Wood G.C. (2013). Concepts and practices used to develop functional PLGA-based na-noparticulate systems. Int. J. Nanomed..

[69] Perrie Y., Kastner E., Kaur R., Wilkinson A., Ingham A.J. (2013). A case-study investigating the physicochemical characteristics that dictate the function of a liposomal adjuvant. Hum. Vaccines Immunother..

[70] Uto T., Akagi T., Toyama M., Nishi Y., Shima F., Akashi M., Baba M. (2011). Comparative activity of biodegradable nanoparticles with aluminum adjuvants: Antigen uptake by dendritic cells and induction of immune response in mice. Immunol. Lett..

[71] Uto T., Wang X., Sato K., Haragushi M., Akagi T., Akashi M., Baba M. (2007). Targeting of antigen to dendritic cells with poly(γ-glutamic acid) nanoparticles induces antigen specific humoral and cellular immunity. J. Immunol..

[72] Uto T., Akagi T., Hamasaki T., Akashi M., Baba M. (2009). Modulation of innate and adaptive immunity by biodegradable nanoparticles. Immunol. Lett..

[73] Wang X., Uto T., Akagi T., Akashi M., Baba M. (2008). Poly(gamma-glutamic acid) nanoparticles as an efficient antigen delivery and adjuvant system: Potential for an AIDS vaccine. J. Med. Virol..

[74] Uto T., Akagi T., Yoshinaga K., Toyama M., Akashi M., Baba M. (2011). The induction of innate and adaptive immunity by bio-degradable poly(γ-glutamic acid) nanoparticles via a TLR4 and MyD88 signalling pathway. Biomaterials.

[75] Shima F., Akagi T., Akashi M. (2015). Effect of hydrophobic side chains in the induction o immune responses by nanoparticle ad-juvants consisting of amphiphilic Poly(γ-glutamic acid). Bioconjug. Chem..

[76] Shima F., Akagi T., Uto T., Akashi M. (2013). Manipulating the antigen-specific immune response by the hydrophobicity of amphiphilic poly-(γ-glutamic acid) nanoparticles. Biomaterials.

[77] Shima F., Uto T., Akagi T., Baba M., Akashi M. (2013). Size effect of amphiphilic poly(γ-glutamic acid) nanoparticles on cellular uptake and maturation of dendritic cells in vivo. Acta Biomater..

[78] Thomann-Harwood L.J., Kaeuper P., Rossi N., Milona P., Herrmann B., McCullough K.C. (2012). Nanpgel vaccines targeting dendritic cells: Contributions of the surface decoration and vaccine cargo on cell targeting and activation. J. Control. Release.

[79] Cruz L.J., Tacken P.J., Fokkink R., Figdor C.G. (2011). The influence of PEG chain length and targeting moiety on anti-body-mediated delivery of nanoparticle vaccines to human dendritic cells. Biomaterials.

[80] de Oliveira C.I., Santos D.M., Carneiro M.W., de Moura T.R., Fukutani K., Clarencio J., Soto M., Espuelas S., Brodskyn C., Barral A. (2012). Towards development of novel immunization strategies against leishmaniasis using PLGA nanoparticles loaded with kinetoplastid membrane protein-11. Int. J. Nanomed..

[81] Han J., Zhao D., Li D., Wang X., Jin Z., Zhao K. (2018). Polymer-Based Nanomaterials and Applications for Vaccines and Drugs. Polymers.

[82] Zhou J., Krishnan N., Jiang Y., Fang R.H., Zhang L. (2021). Nanotechnology for virus treatment. Nano Today.

[83] Van Der Weken H., Cox E., Devriendt B. (2020). Advances in Oral Subunit Vaccine Design. Vaccines.

[84] Al-Halifa S., Gauthier L., Arpin D., Bourgault S., Archambault D. (2019). Nanoparticle-Based Vaccines against Respiratory Viruses. Front. Immunol..

[85] Wibowo D., Jorritsma S.H., Gonzaga Z.J., Evert B., Chen S., Rehm B.H. (2021). Polymeric nanoparticle vaccines to combat emerging and pandemic threats. Biomaterials.

[86] Siegrist S., Cörek E., Detampel P., Sandström J., Wick P., Huwyler J. (2019). Preclinical hazard evaluation strategy for nanomedicines. Nanotoxicology.

[87] Patel P., Shah J. (2018). Safety and Toxicological Considerations of Nanomedicines: The Future Directions. Curr. Clin. Pharmacol..

[88] Swierczewska M., Crist R.M., McNeil S.E. (2017). Evaluating Nanomedicines: Obstacles and Advancements. Adv. Struct. Saf. Stud..

[89] Duncan R. (2017). Polymer therapeutics at a crossroads? Finding the path for improved translation in the twenty-first century. J. Drug Target..

[90] Stojanowski J., Gołębiowski T. (2021). Focus on COVID-19: Antiviral polymers in drugs and vaccines. Polym. Med..

[91] de Queiroz N.M.G.P., Marinho F.V., Chagas M.A., Leite L.C.C., Homan E.J., de Magalhães M.T.Q., Oliveira S.C. (2020). Vaccines for COVID-19: Perspectives from nucleic acid vaccines to BCG as delivery vector system. Microbes Infect..

[92] Silveira M.M., Moreira G.M.S.G., Mendonça M. (2021). DNA vaccines against COVID-19: Perspectives and challenges. Life Sci..

[93] Jiang X., Li Z., Young D.J., Liu M., Wu C., Loh X.J. (2021). Toward the prevention of coronavirus infection: What role can poly-mers play?. Mater. Today Adv..

